# Determination of Oxaliplatin by a UHPLC-MS/MS Method: Application to Pharmacokinetics and Tongue Tissue Distribution Studies in Rats

**DOI:** 10.3390/ph15010052

**Published:** 2021-12-31

**Authors:** Xiuqing Gao, Robert Y. L. Tsai, Jing Ma, Yang Wang, Xiaohua Liu, Dong Liang, Huan Xie

**Affiliations:** 1 Department of Pharmaceutical Science, College of Pharmacy and Health Sciences, Texas Southern University, Houston, TX 77004, USA; xiuqing.gao1990@gmail.com (X.G.); jing.ma@tsu.edu (J.M.); yang.wang@tsu.edu (Y.W.); dong.liang@tsu.edu (D.L.); 2 Department of Translational Medical Sciences, Institute of Biosciences and Technology, Texas A&M Health Science Center, Houston, TX 77030, USA; robtsai@tamu.edu; 3 Department of Biomedical Sciences, Baylor College of Dentistry, Dallas, TX 75246, USA; xliu1@tamu.edu

**Keywords:** oxaliplatin, LC-MS/MS, plasma, tongue tissue, intravenous administration, pharmacokinetics

## Abstract

Oxaliplatin (OXP), a third-generation platinum-based chemotherapy drug, was often indirectly analyzed via total platinum by an ICP-MS because it was difficult to directly quantify using an LC-MS/MS method, due to its instability, bad column separability and severe MS signal inhibition. Here, we developed and validated a specific, sensitive and reproducible LC-MS/MS method for the quantification of OXP itself in rat plasma and tongue tissue on a SCIEX 4000 QTRAP^®^ MS/MS system equipped with a Phenomenex Lux 5u Cellulose-1 column (250 × 4.6 mm, 5 μm). This method was validated at the lower limit of detection (LOD) and the lower limit of quantitation (LLOQ) of 5 ng/mL and 10 ng/mL, with linearity of 10–5000 ng/mL (r^2^ > 0.99) and 10–2500 ng/mL (r^2^ > 0.99), in rat plasma and tongue homogenates, respectively. The intra- and inter-day precision (CV%) and accuracy (RE%) were within 15% for LLOQ, low-, medium- and high-quality control samples. The mean extraction recoveries were around 50% and 80% for plasma and tongue homogenates, respectively. This assay was successfully applied to pharmacokinetics study following intravenous administration of OXP, as well as tongue tissue distribution after 1 h and 4 h of a novel oral mucosal patch application.

## 1. Introduction

**Figure 1:**
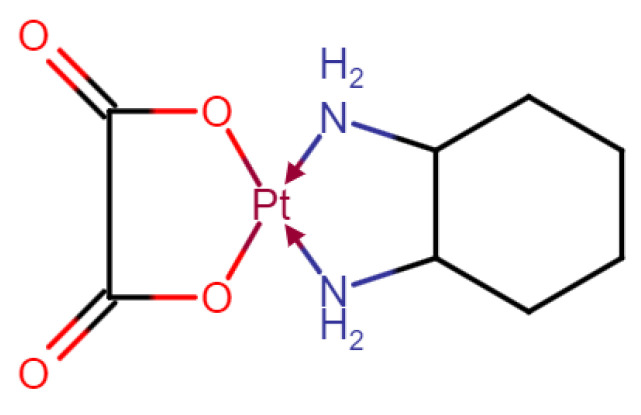
Chemical structure of oxaliplatin (OXP).

Oxaliplatin (OXP, brand name Eloxatin^®^, Figure 1) is a third-generation platinum-based antineoplastic drug widely used for solid tumor treatment in the clinic. It is an analogue of cisplatin by substituting the amino group of cisplatin with diaminocyclohexane (DACH). It was discovered in 1976, and approved by European Medicines Agency (EMA) in 1996 and the U.S. Food and Drug Administration (FDA) in 2002 [1]. Currently, OXP is typically used along with 5-fluorouracil and folinic acid (leucovorin) in a combination known as FOLFOX, a first-line treatment for stage III colorectal cancers. It shows higher anti-tumor activity and less toxicity as compared to cisplatin and carboplatin, due to its 1,2-DACH carrier ligand [2]. Recent studies showed that OXP had strong synergistic inhibition effects on oral squamous cell carcinoma (OSCC-25) cells when used along with mycophenolic acid, making OXP a potential candidate for treating oral precancerous lesions [3,4].

Like previous two generations of platinum-containing drugs, OXP also rapidly forms a variety of reactive intermediates after intravenous administration in vivo [5]. It undergoes rapid and extensive non-enzymatic biotransformation and has no evidence of being metabolized by cytochrome P450 [6]. In patients, there are at least 17 biotransformed products of OXP by reaction with water, chloride, glutathione and methionine [7,8,9,10]. Pt(DACH)Cl_2_, Pt(DACH)Cl(OH), and dihydrated OXP complex (DOC) are thought to be active biotransformation products of OXP. However, those products do not make a significant contribution to the cytotoxicity of OXP even though they are known to be more cytotoxic than OXP in cellular assays [11,12,13]. Conventionally, total free platinum of OXP in plasma ultrafiltrates (PUF) is measured by flame atomic absorption spectroscopy (FAAS) or the more sensitive inductively coupled plasma mass spectrometry (ICP-MS) [14,15]. However, the active fraction decreases with time as OXP is biotransformed to inactive metabolites. Since those inactive biotransformed products of OXP are also included in PUF, the total free platinum concentration is often overestimated in the pharmacokinetics (PK) study of the active components [16]. Furthermore, the parent OXP is the major active platinum complex at least during the first few hours following OXP infusion in humans. Therefore, parent OXP quantification is more important for the PK evaluation, and a sensitive and reliable bioanalytical method for the quantification of parent OXP is critically needed for in vivo studies [17]. Falta et al. [18] have developed a rapid and robust quantification method for oxaliplatin and other cancerostatic platinum compounds (CPC) by ICP- sector field MS (ICP-SFMS) and hydrophilic interaction liquid chromatography (HILIC) combined with ICP quadrupole-base instrument (ICP-QMS) detection. ICP-SFMS was used to quantify the total platinum concentrations from volunteer male subjects’ whole blood compartments, while the HILIC-ICP-QMS method can be used to quantify intact CPC concentrations using CPC spiked plasma samples concentration. However, their method was neither validated nor applied to pharmacokinetics analysis. In addition, since only one third of the oxaliplatin is present as parent compound in unbounded plasma fraction, a more accurate species-specified method is needed.

Oral mucosal drug delivery has unique benefits compared to other routes of administration. It avoids the first-pass effect from liver and gastrointestinal (GI) tract. It also has the potential to reduce systemic side effect with less systemic circulation exposure, to improve patient compliance, to provide fast on-site action and localized delivery, as well as to offer flexible dosing [19]. There are three categories of drug delivery in the oral mucosal cavity: sublingual delivery, buccal delivery, and local delivery [20]. Matos et al. [21] have reported the ex vivo application of mucoadhesive topical treatment in porcine tongue using polymeric OXP nanoparticles and substantiated the feasibility of topical therapy by showing an increase of drug penetration ex vivo. However, no previous studies have investigated the topical administration in oral mucosa in vivo to the best of our knowledge.

To date, there are only two studies that reported parent OXP quantification by liquid chromatography with tandem mass spectrometry (LC-MS/MS) in plasma, with a lower limit of quantification (LLOQ) of 20 and 25 ng/mL [22,23]. In this study, a novel bioanalytical method with a much higher sensitivity (10 ng/mL LLOQ and 5 ng/mL lower limit of detection (LOD)) was developed and validated for the quantification of parent OXP in rat plasma and tongue tissue samples after supralingual administration of a novel OXP patch in rats.

## 2. Results and Discussion

### 2.1. Method Development

#### 2.1.1. Mass Spectrometry

**Figure 2:**
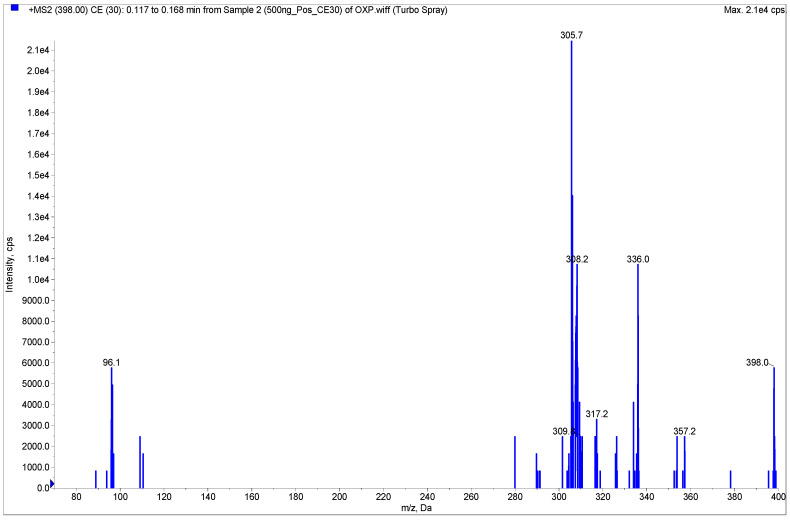
Product ion mass spectra of OXP (*m/z*: 398.06→306.00).

^194^Pt, ^195^Pt, ^196^Pt and ^198^Pt are four major natural isotopes of platinum. Their content ratios in nature are 32.9%, 33.9%, 25.3% and 7.2%, respectively. Thus, positive ion full scan mass spectra (Q1) of OXP molecular ion [M + H]^+^ exhibited *m/z* values of 397, 398, 399 and 401 [22]. The strongest *m/z* 398 was chosen as the precursor ion. The electrospray ionization (ESI) mode resulted in lower noise background and better signal intensities for both OXP and IS. Multiple reaction monitoring (MRM) mode was used to identify the molecules by monitoring the transition *m/z* 398.1→306.0 for OXP and *m/z* 189.0→131.0 for the IS. For the OXP, *m/z* 306 is generated by the loss of two HCOOH molecules from protonated molecular ions ([M + H]^+^-2HCOOH). For the IS, *m/z* 131, a stable conjugated π bond system [CH=CH-N=N-C_6_H_5_]^+^, is formed after the loss of a molecule of CH_3_COCH_3_. Those daughter ions were stable to be used in quantification. Since stable isotope-labeled OXP was not available on the market, antipyrine was chosen as an IS based on its appropriate retention time and no interference in this method. The product ion mass spectra for OXP is shown in Figure 2. Instrument MS source- and compound-dependent parameters were optimized by tuning to improve OXP sensitivity. The method was validated using these optimized conditions as described in the method.

#### 2.1.2. Chromatography Separation

**Figure 3:**
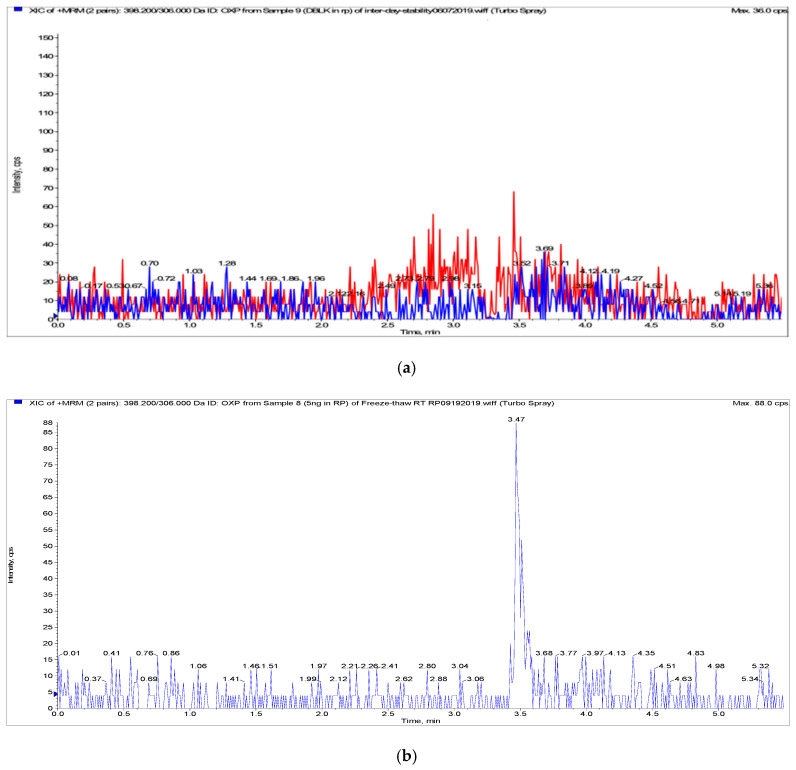

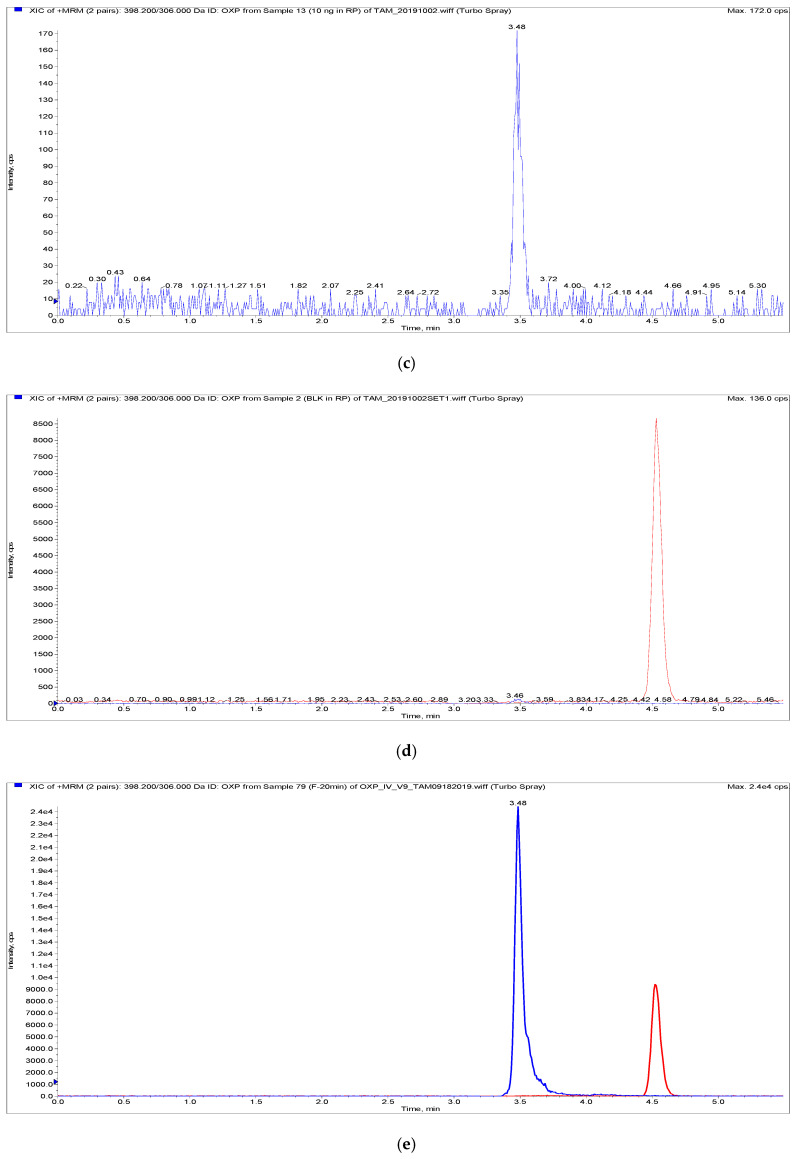
Representative chromatograms of OXP and internal standard (IS) in blank and spiked rat plasma: (**a**) blank plasma, (**b**) OXP spiked in plasma at the lower limit of detection (5 ng/mL), (**c**) OXP spiked in plasma at the lower limit of quantification (10 ng/mL), (**d**) blank plasma spiked with IS, (**e**) rat plasma sample at 20 min after 25 mg/kg body weight (BW) intravenous (IV) administration. OXP transition depicted in blue and IS transition depicted in red.

**Figure 4:**
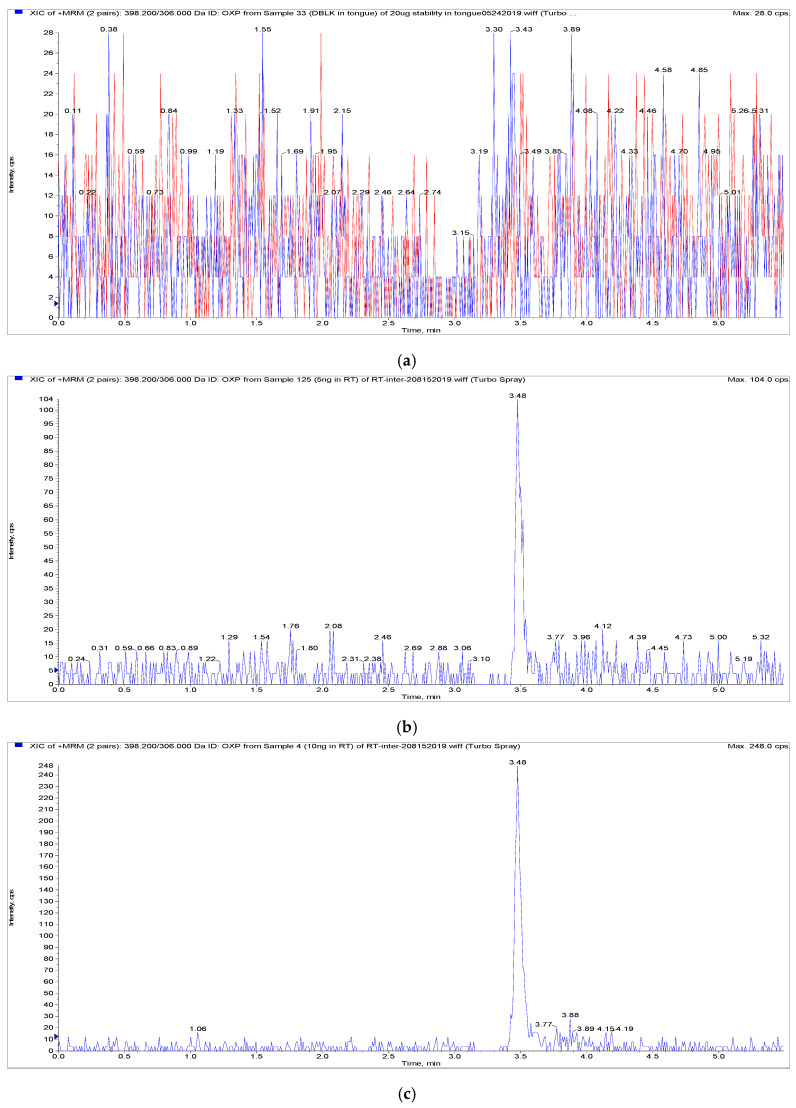

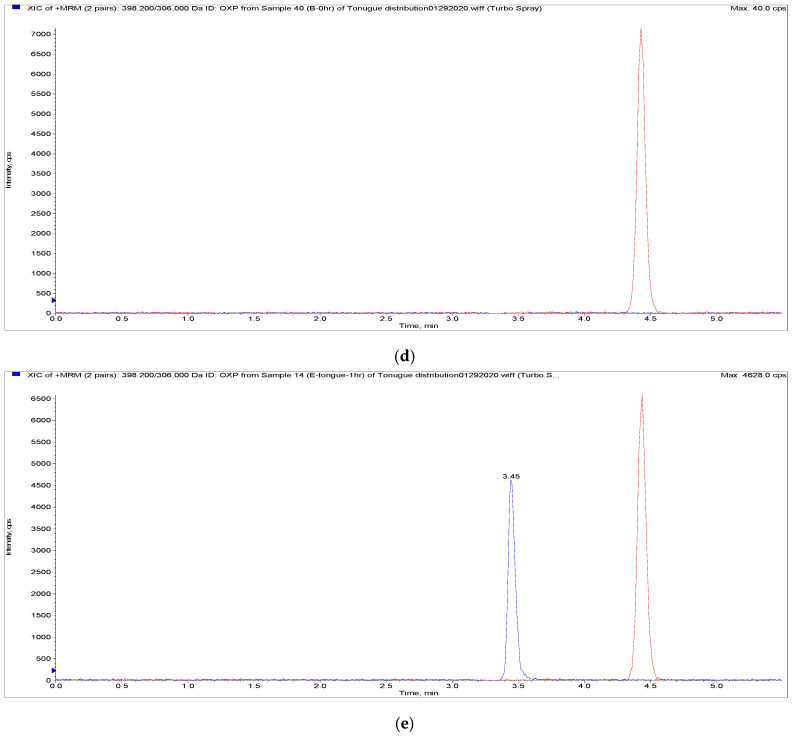
Representative chromatograms of OXP and IS in blank and spiked tongue homogenates: (**a**) blank tongue homogenates, (**b**) OXP spiked in tongue homogenates at the lower limit of detection (5 ng/mL), (**c**) OXP spiked in tongue homogenates at the lower limit of quantification (10 ng/mL), (**d**) blank tongue homogenates spiked with internal standard, (**e**) rat tongue sample at 1 h following patch application. OXP transition depicted in blue and IS transition depicted in red.

OXP and internal standard (IS) were separated on a Phenomenex Lux 5u Cellulose-1 column (250 × 4.6 mm, 5 μm). Several other short stationary phase columns were tested, including the Kinetex^®^ F5 column (50 × 2.1 mm, 1.7 μm), Acquity HSS-T3 (50 × 2.1 mm, 1.8 μm) and ACE Excel 2 Super C_18_ column (50 × 2.1 mm, 2 μm), and Phenomenex Lux 5u Cellulose-1 column (100 × 4.6 mm, 5 μm). However, all of them showed serious signal inhibition for plasma samples, suggesting that the OXP signal might be interfered with by endogenous substances. With a longer Phenomenex Lux 5u Cellulose-1 column, the simple isocratic elution could separate OXP and IS more efficiently than the other columns, where retention times of OXP and IS were 3.48 min and 4.54 min, respectively. Notably, 1.25 mM of ammonia formate was needed for the mobile phase to obtain consistent signal, indicating that the pH of mobile phase could affect the quality of the LC-MS/MS analyzing method. Different pH of mobile phase has been investigated, it being observed that adding 0.05% and 0.1% of formic acid to mobile phase under same condition could lead to double peak at high concentration, and the neutral mobile phase without adding acidic or alkaline solution could lead to unstable sensitivity of OXP. The final UHPLC method was optimized by selecting and testing different types of column, mobile phase compositions and flow rates to obtain better peak shapes, less carryover and higher sensitivity. As shown in Figure 3 and Figure 4, IS had no interference with OXP. There was also no carryover detected in either blank plasma or tongue homogenate samples after six injections of ULOQ by a simple needle wash method. By comparing our method to the previous reported HILIC-ICP-QMS method [18], we gain advantages by using a simple isocratic elution method instead of a gradient elution method with shorter retention time (3.50 min verse 6.9 min).

### 2.2. Method Validation

#### 2.2.1. Selectivity and Specificity

As shown in Figure 3 and Figure 4, there was no interference or significant ion suppression detected from endogenous matrix components, and there was no carryover for either IS (≤5% of average response) or OXP (≤20% of LLOQ), which meets the FDA’s bioanalytical guidelines [24].

#### 2.2.2. Sensitivity and Linearity

The standard curve showed good linearity from 10 ng/mL to 5000 ng/mL for plasma and 10 ng/mL to 2500 ng/mL for tongue homogenates. Linear correlation coefficients (r^2^) were at least 0.99 (e.g., y = 0.00088x + 0.00125 with r^2^ = 1.0000 for rat plasma; y = 0.00146x + 0.00634 with r^2^ = 0.9994 for tongue homogenates) for all calibration curves. The accuracy was within 85–115% for plasma and tongue homogenate calibration standards at all concentration levels. LOD of the assay was 5 ng/mL and LLOQ was 10 ng/mL for both plasma and tongue homogenates, where LOD had at least 3:1 and LLOQ had at least 5:1 signal-to-noise ratio (Figure 3 and Figure 4). Lower LOD and LLOQ levels have been achieved with a simpler sample preparation than previous studies by LC-MS/MS (LLOQ: 20 and 25 ng/mL) and by HILIC-ICP-QMS (LLOQ: 40 ng/mL) [18,22,23].

#### 2.2.3. Accuracy and Precision

The inter-day and intra-day accuracy and precision were determined at the LLOQ, LQC (low quality control), MQC (medium quality control), HQC (high quality control) in rat plasma and tongue homogenates with five replicates (Table 1). The intra-day and inter-day accuracy (relative error = RE%) ranged from −6.70% to 4.21% and −4.68% to 4.48% in the plasma and tongue homogenates, respectively. The precision (coefficient of variation = CV%) ranged from 2.96% to 8.67% and 2.37% to 7.88% in the plasma and tongue homogenates, respectively. The precision and accuracy were within the acceptance range according to FDA bioanalysis guidance. These results suggest that OXP in the plasma and tongue homogenates can be measured accurately and reproducibly by the present method.

#### 2.2.4. Dilution Integrity

A dilution integrity study was performed to check if sample dilution changed signals compared to the predicting concentration. All of them were within ±15% of nominal concentration, indicating that the dilution was reliable for quantification of samples with higher OXP concentration than ULOQ.

#### 2.2.5. Matrix Effect and Extraction Recovery

The extraction recovery and matrix effect from different biological matrices are shown in Table 2. The matrix effects in the plasma ranged from 37.17% to 47.02% and the recovery was around 50%, which implied that plasma contents significantly suppressed the signal and decreased the extraction efficiency for OXP, and it might explain why it was difficult to increase sensitivity as reported by previous studies. Matrix effects might be reduced by diluting samples with more acetonitrile. However, the sensitivity of OXP should be considered. Matrix effects in tongue homogenates were all less than 15% and the recovery ranged from 80.12% to 86.36%, suggesting a negligible matrix effect and good extraction efficiency in tongue homogenates.

#### 2.2.6. Stability

OXP stability in rat plasma and tongue homogenates was tested for short-term stability, freeze-thaw stability, auto-sampler stability, and long-term stability at four QC levels with triplicates (Table 3 and Table 4). Bench-top stability, performed at 4 °C for 6 h, was with acceptable CV% and RE% (within 15%), indicating that the samples were stable under the laboratory handling condition. QCs in auto-sampler were also stable for 6 h, demonstrating the stability of extracts throughout the process. There were 36.38% loss of OXP in rat plasma and 45.34% loss in rat tongue samples after three freeze-thaw cycles. In contrast, less than 15% of OXP loss with acceptable CV% and RE% were achieved after one and two cycles of freeze–thaw in both rat plasma and tongue homogenates. These results indicate that, after rat plasma and tongue samples are collected from PK and tissue distribution study, samples should be divided into several tubes before −80 °C storage to avoid frequent freeze–thaws, and should be analyzed within 7 days, as a significant loss of OXP is expected after 30 days of storage even at −80 °C. Stock solutions for OXP and IS are stable at 4 °C for up to 6 months.

### 2.3. Pharmacokinetics and Tongue Distribution Study

**Figure 5:**
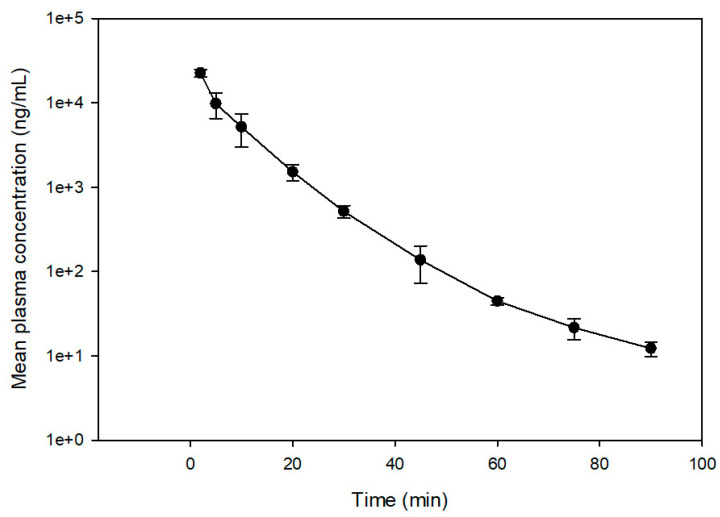
The profiles of the mean ± SD plasma concentration versus time after 25 mg/kg BW OXP intravenous injection to rats (*n* = 3).

No noticeable signs of discomfort were observed in rats after IV administration of OXP (25 mg/kg BW) in 5% glucose solution. The mean plasma concentration versus time profile is shown in Figure 5. Plasma concentration of OXP at 2 h after administration was lower than the LLOQ. The main PK parameters for parent OXP were calculated using non-compartmental analysis [25,26]. Our results showed that the mean ± SD plasma concentration was 22,600 ± 2400 ng/mL at 2 min after IV administration. The mean plasma concentration–time curve during the period of observation (AUC_0→90min_) was 200.4 ± 35.6 min∙μg/mL. The apparent volume of distribution (V_d_) was 867.0 ± 75.3 mL/kg, the apparent clearance (CL) was 127.8 ± 25.2 mL/min/kg, and total body mean residence time (MRT) and terminal elimination half-life (T_1/2_) were 6.9 ± 0.7 and 16.2 ± 2.5 min, respectively (Table 5). The findings are comparable to previously reported rat PK for parent OXP [27,28], supporting the applicability of this method in a pre-clinical PK study of OXP.

**Figure 6:**
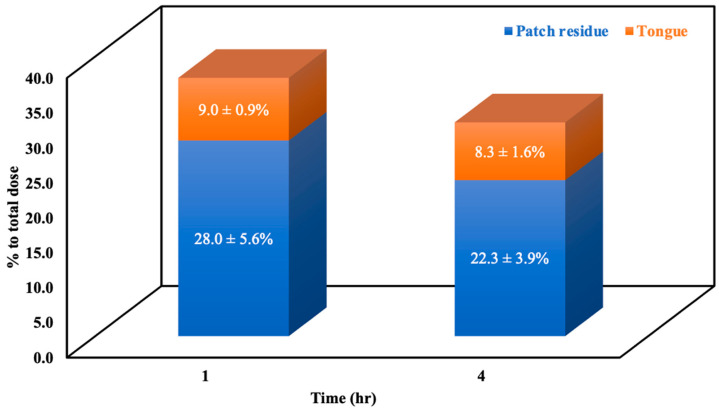
Patch residue and tongue tissue concentration percentage to total dosing of OXP by supralingual administration.

The OXP concentration in plasma after patch administration was lower than LOD. We determined the concentration of OXP in tongue homogenates at 1 h and 4 h following patch application, the mean ± SD of tongue tissue concentrations were 2620 ± 432 ng/g and 2834 ± 664 ng/g, respectively. The percentage values of OXP in patch residue and tongue tissues at 1 h and 4 h after supra-lingual application are presented in Figure 6. Around 28% and 22% of OXP remained in the patch formulation at 1 h and 4 h, respectively. About 9% and 8% of OXP were accumulated inside tongue tissues at 1 h and 4 h after patch application, respectively. OXP tongue concentrations at 1 h and 4 h are similar, indicating that supralingual delivery can lead to long-term absorption and accumulation of OXP inside of the tongue tissue. The OXP patch may lead to a burst release within the first 1 h followed by a long-term sustained release [20]. In addition, our previous study on drug mycophenolic acid showed relatively lower plasma exposure in systemic circulation and higher accumulation in tongue tissue after supraglingual administration, suggesting that supralingual patch delivery may provide the benefits of longer drug accumulation in the tongue tissues and less systemic exposure [29].

Several drug dosage formulations have been reported for intraoral application, including the implantable tablet, mucoadhesive patch, film, microsphere, ointment, cream, and hydrogel [30,31,32,33,34,35]. Most of these applications were instantaneously dissolved with a rapid onset, especially when administered at the non-keratinized sublingual and buccal surface [36,37]. The dorsal tongue has both non-keratinized and keratinized area, making it ideal for both local and systemic delivery [38,39]. Also, polyacrylic acid-974 (PAA) and carboxymethyl cellulose (CMC) are effective anionic polymers to maintain patch adhesion to oral mucosa [40]. Using a polymer-based patch formulation for topical drug delivery allows a sustained drug release [41]. In summary, the application of an OXP polymeric patch to dorsal tongue represented a novel study and warrants further investigation as a new therapeutic modality for intraoral lesions.

## 3. Materials and Methods

### 3.1. Chemicals and Reagents

OXP, antipyrine (as internal standard, IS), and ammonia formate were purchased from Sigma Aldrich (St. Louis, MO, USA). Acetonitrile and high-performance liquid chromatography (HPLC)-grade water were obtained from VWR Chemicals BDH (Chicago, IL, USA). Blank rat plasma used for the method’s development and validation was purchased from Innovative Research (Novi, MI, USA).

### 3.2. Animal Purchase and Procedures

Adult male Sprague-Dawley rats were used in all animal studies and were purchased from Envigo RMS (Indianapolis, IN, USA) and tissues were from the non-treated rats. The animal experiments were approved (Protocol #9080) by the Institutional Animal Care and Use Committee (IACUC) at Texas Southern University (TSU) and were conducted according to the National Institute of Health “Guide for the Care and Use of Laboratory Animals, 8th Edition”.

### 3.3. Instruments and Conditions

The UHPLC-MS/MS system includes a Shimadzu Nexera X2 UHPLC system (Columbia, MD, USA) and a 4000 QTRAP^®^ MS/MS system (AB Sciex, Redwood City, CA, USA). System control and data analysis were performed using the Analyst^®^ software 1.6.2 (Sciex, Redwood City, CA, USA).

#### 3.3.1. Ultra High-Performance Liquid Chromatography (UHPLC) Conditions

OXP separation was carried out on a Phenomenex Lux 5u Cellulose-1 column (250 × 4.6 mm, 5 μm) with an isocratic elution of 50% (*v/v*) acetonitrile in water containing 1.25 mM ammonia formate. The injection volume of each sample was 10 μL. The total run time was 5.5 min. The flow rate was 0.8 mL/min.

#### 3.3.2. Tandem Mass Spectrometry (MS/MS) Conditions

MRM data were collected by the MS using a Turbo V™ source with ESI positive mode to detect the specific precursor-to-product ion transitions, *m/z* 398.1→306.0 for OXP and *m/z* 189.0→131.0 for IS antipyrine. The ion spray voltage was set at 5000 V, and the source temperature was set at 700 °C. The pressures of nebulizer gas and heater gas were 60 psi and 55 psi, respectively. The optimized curtain gas pressure was 25 psi and high collision gas “CAD” pressure was applied. Compound-dependent parameters for OXP and IS were optimized and set at 74 and 60 V for declustering potential (DP), 39 and 28 V for collision energy (CE), and 13 and 9 V for collision cell exit potential (CXP), respectively.

### 3.4. Preparation of Stock Solutions, Calibration Standards and Quality Controls

Stock solutions of OXP and IS were dissolved in water at a concentration of 1 mg/mL and stored at 4 °C until use. Standard samples were prepared by spiking OXP into blank rat plasma and rat tongue homogenates, respectively, at concentrations ranging 10–5000 ng/mL and 10–2500 ng/mL for rat plasma and tongue homogenates, respectively. Briefly, a series of working solutions of OXP were prepared by diluting stock solution in water to the appropriate concentrations (10 times as that of nominal concentration) for standards (100, 500, 2500, 5000, 10,000, 25,000, 50,000 ng/mL) in plasma and tissue homogenates. Working solutions were then spiked into blank rat plasma and blank tissue homogenates with 10 times dilution to make calibration standards. The final concentrations of standard samples were 10, 50, 250, 500, 1000, 2500, 5000 ng/mL in plasma. The final concentrations of calibration standards for tongue homogenates were 10, 50, 250, 500, 1000, 2500 ng/mL in tissue homogenates. Calibration standards were freshly prepared daily.

The working solutions for QC samples were independently prepared by diluting OXP standard solutions with water to give the concentrations of 100, 250, 20,000, and 40,000 ng/mL, which were 10 times as that of nominal concentrations: 10 (LLOQ), 25 (LQC), 2000 (MQC) and 4000 ng/mL (HQC) for rat plasma. For tongue homogenates, the working solution concentrations of QC samples were 100, 250, 8000, and 20,000 ng/mL, and the corresponding nominal concentrations were 10 (LLOQ), 25 (LQC), 800 (MQC) and 2000 ng/mL (HQC).

### 3.5. Extraction of OXP from Plasma and Tongue Samples

Each weighted tongue tissue sample was homogenized in a clean scintillation vial with water (1:6, *w/v*) using the Biospec Tissue TearorTM (Bartlesville, OK, USA) to make tongue homogenates. Rat plasma or tongue homogenates were extracted by a protein precipitation method described in [4]. Briefly, 10 μL of 100 ng/mL IS was added to 50 μL of rat plasma or tongue homogenates samples. Next, the samples were extracted by 200 μL of acetonitrile containing 0.01% (*w/v*) ammonium formate. After vortexing for 15 s, the extracted samples were centrifuged at 14,000 rpm for 20 min at 4 °C. Finally, 10 μL of supernatant was injected into the LC-MS/MS system. Sample preparation was performed on top of ice pads.

### 3.6. Method Validation

The assay on rat plasma and tongue sample homogenates were validated following the FDA and the EMA guidelines for bioanalytical method validation with specific aspects described below [24,42].

#### 3.6.1. Selectivity and Specificity

The selectivity and specificity tests were conducted by comparing chromatograms of six different endogenous sources of blank rat plasma or tongue homogenates for interference with the analyte and IS. The peak response of the endogenous plasma and tongue homogenates for the analytes should be ≤20% of the peak area of the LLOQ standard, the IS should be ≤5% of the average peak area of the standard curve and QC samples.

#### 3.6.2. Sensitivity and Linearity

The concentrations of OXP in unknown samples were calculated using linear calibration curves in plasma or tongue homogenates by plotting the peak area of the OXP-to-IS ratio against known standard concentrations of OXP. The slope, intercept, and coefficient of determination were estimated using least squares linear regression method with a weighting of “1/x”. The LOD was selected based on a signal-to-noise ratio of 3:1 and the LLOQ was estimated based on the signal-to-noise ratio of at least 5:1.

#### 3.6.3. Carryover

Triplicate injections of blank samples were conducted following six consecutive injections of QC samples with the highest amounts (ULOQ = 5000 ng/mL for plasma, 2500 ng/mL for tongue homogenates). Carryover in the blank sample following the ULOQ should not be greater than 20% of the LLOQ for OXP and 5% for the IS.

#### 3.6.4. Accuracy and Precision

Intra-day accuracy and precision were conducted at four QC levels of OXP concentrations in plasma and tongue homogenates on a single assay by five replicates. Inter-day accuracy and precision were determined by five replicates of four QC levels on three consecutive days. Accuracy was determined by the RE%. Precision was determined by the CV%. Calculated results of inter-day and intra-day precision and accuracy should be ≤15%, except for the LLOQ, where the absolute value of calculated concentrations should be ≤20%.

#### 3.6.5. Dilution Integrity

The dilution integrity of rat plasma performed to determine the accuracy of extended linearity beyond the ULOQ (5000 ng/mL). The dilution factors for dilutions of QC were 2, 5 and 10. Diluted quality control (DQC, 20 μg/mL) samples were further diluted five and 10 times using plasma to measure the accuracy and precision of the dilution. Another DQC (10 μg/mL) plasma sample was further diluted two and five times to measure the accuracy and precision of the dilution.

#### 3.6.6. Extraction Recovery and Matrix Effect

In this study, matrix effects were evaluated by comparing the peak area of the post-extracted blank rat plasma or tongue homogenates to the peak area of neat solution at four QC levels (LLOQ, LQC, MQC, HQC) in five replicates. The calculated equation of the matrix effect is as follows:

(1) $$\mathrm{Matrix}\;\mathrm{effect}\%=\left[\left(\frac{{\mathrm{Response}\;}_{\mathrm{post}\text{-}\mathrm{extraction}\;\mathrm{spiked}\;\mathrm{samples}}}{{\mathrm{Response}}_{\mathrm{neat}\;\mathrm{solution}\;\mathrm{samples}}}\right)-1\right]\times 100$$

The extraction recovery was evaluated by comparing the peak area of the pre-extracted blank rat plasma or tongue QC sets to the peak area of the post-extracted QC sets. The calculated equation of extraction recovery is as follows:

(2) $$\mathrm{Extraction}\;\mathrm{recovery}\%=\;\left(\frac{{\mathrm{Response}\;}_{\mathrm{pre}\text{-}\mathrm{extraction}\;\mathrm{spiked}\;\mathrm{samples}}}{{\mathrm{Response}}_{\mathrm{post}\text{-}\mathrm{extraction}\;\mathrm{spiked}\;\mathrm{samples}}}\right)\times 100$$

#### 3.6.7. Stability

The stability of OXP in rat plasma or tongue homogenates was examined by short-terms, freeze-thaw, post-prepared auto-sampler, and long-term stability studies. All stability experiments were conducted at four QC levels (LLOQ, LQC, MQC, HQC) with five replicates. The short-term bench-top stability was tested by placing prepared QC samples at 4 °C for 6 h. Auto-sampler stability was performed by placing QC samples inside of autosampler of LC-MS/MS for 6 h, the auto-sampler temperature was set to 15 °C. Freeze-thaw stability was conducted by three cycles of freezing at −80 °C and thawing at 4 °C. Long-term stability was evaluated by placing the QC samples in −80 °C. OXP concentrations in all stability samples were compared to that of freshly made QC samples.

### 3.7. Patch Formulation

The polymer patches were fabricated by Cellink Bio X using a high-OXP-concentration solution containing 30 mg of OXP, 0.05 g of PAA, 0.05 g of 3,4-dihydroxyphenylalanine (DOPA), 0.1 g of CMC, and 5 mL of H_2_O, on a supporting material. The concentration of OXP in each patch was 1.1 μg/cm^2^.

### 3.8. Pharmacokinetics and Tongue Distribution Studies

The validated method was applied to investigate the plasma profiles of OXP after IV administration of a 25 mg/kg BW OXP in 5% glucose solution, and tongue distribution of OXP after a supralingual administration of 150 μg OXP patch/kg BW in rats. A group of three adult male SD rats was used for the PK studies. Serial blood samples (approximately 150 μL for each) were collected in heparinized tubes at various time points up to 24 h after dosing. Plasma was separated immediately by centrifugation of the blood samples at 3000 rpm for 20 min and kept at −80 °C until analysis. For the tongue tissue distribution and blood diffusion studies, six adult male SD rats were used for the study. A mucoadhesive patch formulation containing OXP was applied onto the dorsal tongue surface of a rat for 1 h (*n* = 3) and 4 h (*n* = 3), respectively, at a dose of 150 μg/kg BW under anesthesia. Serial blood samples (about 100 μL each) were collected at 1, 2, 3 and 4 h from the start of the patch application to the end of patch application and stored in heparinized tubes. Blood samples were centrifuged at 3000 rpm for 20 min to separate the plasma samples, then stored at −80 °C until analysis. Tongues were removed immediately after euthanasia, rinsed with deionized water three times, sliced into small pieces, and then homogenized in water (1:6 *w/v*). Blank tongues were prepared separately using drug-free rats. The PK parameters for each rat were estimated using the Phoenix WinNonlin software 8.2 (Certara, NJ, USA). Non-compartmental analysis was used to determine the PK parameters of OXP after IV administration [43].

## 4. Conclusions

In this study, we developed a rapid, sensitive, and reliable UHPLC-MS/MS method for measuring OXP in rat plasma and tongue homogenates. This method was properly validated over the concentration ranges of 10–2500 ng/mL and 10–5000 ng/mL for tongue homogenates and plasma, respectively. We have successfully applied this method to the PK study after IV administration of OXP and showed that the plasma concentration was quantifiable until 1.5 h after administration. Topical delivery of OXP to rat tongue tissue was also determined and showed similar tongue concentrations of OXP at 1 h and 4 h after patch application, indicating a burst penetration of OXP into tongue tissue before 1 h and a long-term sustained release until 4 h of the application.

## Figures and Tables

**Table 1:** Intra-day and inter-day accuracy and precision of OXP in rat plasma and tongue homogenates.

	QCs (ng/mL)	Intra-Day (*n* = 5)			Inter-Day (*n* = 15)		
		Observed Concentration (Mean ± SD)	RE%	CV%	Observed Concentration (Mean ± SD)	RE%	CV%
Plasma	10	9.94 ± 0.85	−0.63	8.54	9.84 ± 0.42	−1.68	4.32
	25	24.86 ± 2.15	−0.58	8.67	26.10 ± 1.09	4.21	4.19
	2000	1874.44 ± 87.40	−6.70	4.66	1903.33 ± 70.05	−5.08	3.68
	4000	3985.00 ± 249.55	−0.38	6.26	4065.00 ± 120.12	1.60	2.96
Tongue	10	10.43 ± 0.44	4.15	4.19	10.45 ± 0.54	4.48	5.17
	25	23.88 ± 0.95	−4.68	3.97	24.95 ± 1.97	−0.20	7.88
	800	783.33 ± 52.95	−2.13	6.76	798.22 ± 49.90	−0.22	6.25
	2000	2000 ± 47.33	0.00	2.37	2059.44 ± 109.52	2.97	5.32

SD, standard deviation; CV, coefficient of variation; RE, relative error, QCs = quality controls.

**Table 2:** Recovery and matrix effect of OXP in rat plasma and tongue homogenates quality control (QC) samples.

Biological Samples	Nominal Concentration (ng/mL)	Matrix Effect (%)	Recovery (%)
		(*n* = 6)	(*n* = 6)
**Plasma**	10	47.02 ± 2.32	51.89 ± 1.50
	25	37.17 ± 3.09	53.44 ± 3.98
	2000	46.61 ± 2.06	55.60 ± 2.24
	4000	43.24 ± 3.14	52.80 ± 6.85
**Tongue**	10	12.43 ± 6.55	86.36 ± 7.00
	25	1.26 ± 9.25	80.12 ± 7.38
	800	0.18 ± 6.36	82.30 ± 5.99
	2000	2.71 ± 6.71	84.32 ± 2.81

**Table 3:** Stability data for OXP in rat plasma.

	Nominal Concentration (ng/mL)	Calculated Concentration (ng/mL)	Precision	Accuracy
		Mean ± SD	CV%	RE%
auto-sampler (6 h) 15 °C	10	9.58 ± 0.48	4.98%	−4.25%
	25	25.15 ± 2.40	9.56%	0.60%
	2000	1937.50 ± 90.69	4.68%	−3.13%
	4000	3487.50 ± 81.80	2.35%	−12.81%
short-term (6 h) 4 °C	10	9.91 ± 0.97	9.77%	−0.95%
	25	23.70 ± 1.61	6.81%	−5.20%
	2000	2052.50 ± 226.62	11.04%	2.63%
	4000	3577.50 ± 102.10	2.85%	−10.56%
1-cycle-freeze thaw −80 °C to RT	10	9.46 ± 0.69	7.28%	−5.43%
	25	23.45 ± 1.25	5.33%	−6.20%
	2000	1950.00 ± 133.42	6.84%	−2.50%
	4000	3985.00 ± 270.62	6.79%	−0.38%
2-cycle-freeze thaw −80 °C to RT	10	8.84 ± 0.15	1.70%	−11.63%
	25	24.00 ± 0.94	3.91%	−4.00%
	2000	1827.50 ± 126.06	6.90%	−8.63%
	4000	3760.00 ± 194.42	5.17%	−6.00%
3-cycle-freeze thaw −80 °C to RT	10	8.93 ± 1.06	11.90%	−10.73%
	25	17.55 ± 4.70	26.81%	−29.80%
	2000	1272.50 ± 250.65	16.16%	−36.38%
	4000	2630.00 ± 351.66	13.37%	−34.25%
Long-term (one month) −80 °C	10	7.185 ± 0.50	6.90%	−28.15%
	25	18.775 ± 0.92	4.89%	−24.90%
	2000	1490 ± 73.94	4.96%	−25.50%
	4000	2990 ± 47.61	1.59%	−25.25%
Long-term (one week) −80 °C	10	9.74 ± 0.21	2.19%	−2.60%
	25	26.9 ± 0.48	1.77%	7.60%
	2000	2127.5 ± 113.25	5.32%	6.38%
	4000	4387.5 ± 195.34	4.45%	9.69%

SD, standard deviation; CV, coefficient of variation; RE, relative error.

**Table 4:** Stability data for OXP in tongue homogenates.

	Nominal Concentration (ng/mL)	Calculated Concentration (ng/mL)	Precision	Accuracy
		Mean ± SD	CV (%)	RE (%)
auto-sampler (6 h) 15 °C	10	9.02 ± 0.72	8.02%	−9.80%
	25	22.40 ± 0.42	1.89%	−10.40%
	800	733.25 ±37.43	5.10%	−8.34%
	2000	1930.00 ± 29.16	2.03%	−3.50%
short-term (6 h) 4 °C	10	10.23 ± 1.43	13.97%	2.25%
	25	26.80 ± 0.85	3.17%	7.20%
	800	858.00 ± 62.06	7.23%	7.25%
	2000	2055.00 ± 59.72	2.91%	2.75%
1-cycle-freeze thaw −80 °C to RT	10	10.875 ± 0.26	2.42%	8.75%
	25	26.6 ± 0.28	1.06%	6.40%
	800	812.5 ± 79.33	9.76%	1.56%
	2000	1970 ± 212.60	10.79%	−1.50%
2-cycle-freeze thaw −80 °C to RT	10	11.025 ± 0.59	5.31%	10.25%
	25	25.05 ± 0.78	3.11%	0.20%
	800	696.00 ± 13.66	1.96%	−13.00%
	2000	1855.00 ± 59.16	3.19%	−7.25%
3-cycle-freeze thaw −80 °C to RT	10	6.57 ± 0.29	4.43%	−34.35%
	25	15.70 ± 0.99	6.31%	−37.20%
	800	437.25 ± 67.07	15.34%	−45.34%
	2000	1097.50 ± 20.62	1.88%	−45.13%
Long-term (one month) −80 °C	10	9.96 ± 0.75	7.49%	−0.45%
	25	20.60 ± 1.27	6.18%	−17.60%
	800	507.75 ± 16.01	3.15%	−36.53%
	2000	1232.50 ± 90.32	7.33%	−38.38%
Long-term (one week) −80 °C	10	9.50 ± 0.35	3.71%	−5.05%
	25	25.25 ± 3.46	13.72%	1.00%
	800	731.25 ± 12.84	1.76%	−8.59%
	2000	2045.00 ± 186.28	9.11%	2.25%

SD, standard deviation; CV, coefficient of variation; RE, relative error.

**Table 5:** Pharmacokinetics parameters of 25 mg/kg BW OXP after intravenous administration to rats (*n* = 3).

		Rat A	Rat B	Rat C	Mean	SD	CV%
Parameter	Units	Estimate					
AUC_0→90min_	min.μg/mL	159.40	217.62	224.09	200.37	35.63	17.8
CL	mL/min/kg	156.84	114.88	111.56	127.76	25.24	19.8
T_1/2_	min	16.85	13.50	18.27	16.21	2.45	15.1
MRT	min	6.06	7.38	7.20	6.88	0.72	10.4
V_d_	mL/kg	949.91	848.24	802.80	866.98	75.32	8.7

SD, standard deviation; CV, coefficient of variation; AUC_0→90min_, area under curve during 90 min; CL, clearance; T_1/2_, half-life; MRT, mean residence time; V_d_, volume of distribution.

## Data Availability

The data has been presented in main text.

## Abbreviations

AUC_0→90min_	plasma concentration–time curve during the period of observation
BW	body weight
CAD	collision gas
CE	collision energy
CL	clearance
CMC	carboxymethyl cellulose
CPC	cancerostatic platinum compounds
CV%	coefficient of variation
CXP	cell exit potential
DACH	diaminocyclohexane
DOC	dihydrated OXP complex
DOPA	3,4-dihydroxyphenylalanine
DP	declustering potential
DQC	diluted quality control
EMA	European Medicines Agency
ESI	electrospray ionization
FAAS	flame atomic absorption spectroscopy
FDA	Food and Drug Administration
FOLFOX	chemotherapy regimen combined 5-fluorouracil, folic acid, and oxaliplatin
GI	gastrointestinal
HILIC	hydrophilic interaction liquid chromatography
HPLC	high-performance liquid chromatography
ICP-MS	Inductively Coupled Plasma Mass Spectrometry
ICP-QMS	ICP quadrupole-based instrument
ICP-SFMS	ICP-sector field MS
IS	internal standard
LC-MS/MS	liquid chromatography with tandem mass spectrometry
LLOQ	lower limit of quantification
LOD	lower limit of detection
MRM	multiple reaction monitoring
MRT	mean residence time
OSCC-25	oral squamous cell carcinoma
OXP	oxaliplatin
PAA	polyacrylic acid-974
PK	pharmacokinetics
PUF	plasma ultrafiltrates
QC	quality control
RE%	relative error
SD	standard deviation
T_1/2_	half-life
V_d_	volume of distribution
